# Portfolio of a Physician

**Published:** 1809-11

**Authors:** 


### PORTFOLIO OF A PHYSICIAN.

### Dietetics. *On Bread, Yeast, &c.*

### *Soluble and digestive Quality of Pulp or Meal, of farinaceous Seeds.*

According to Spallanzani, Goss, Fordyce, and others, we find, among the substances with which the gastric juice becomes soluble, or readily enters into digestion, and which are reducible to a pulp in an hour, or an hour and an half, are of the vegetable tribe, pulp, or the meal of farinaceous seeds; different sorts of wheaten bread without butter, but the crust more than the crumb, and especially the second day after baking. The salted bread of *Geneva* more so, than that of *Paris* without salt. Brown bread, in proportion as it contains more bran, is lesss soluble. But in all cases of digestion, from the nature of the gastric juice, when the stomach is in health, so as to secrete it in a state of purity, the food is very readily digested, unless when in too large a quantity, or of a species which the stomach has not been accustomed to; in this case, the vegetable part will go into a state of acetous fermentation; heart-burn (cardialgia), vomiting, &c. are produced. The writer has known this especially the case with such as have eat heartily of oat cake, (such as is made in the West Riding of Yorkshire, called Havre-cake) for the first time. It is well known that certain fish will produce the same effect upon particular stomachs; but when animal matter is the cause, it must be noted, that the putrid fermentation is always the consequence. We know a gentleman who has always these symptoms if he eat a few raspberries, or if the smallest proportion of this fruit or juice be mixed with others for pastry, though the quantity should be so minute as not to be discernible by the taste. Putridity being after this manner occasion-

 ed ed by animal matter, either by its action on the stomach, or its introduction into the system along with the chyle, has often produced malignant fever. "*Multa huc pertinentia in scriptis medicorum reperiuntur, quæ sub silentio præteribo.*"

The Rev. Mr. James Cooke, so respectably known for his various improvements in husbandry and agricultural implements, recommends yeast to be preserved by coating a board with a whiting brush; allowing the coat to dry; then putting on another, which is in like manner to dry, and so a third, and any number of successive coatings. He assures us, that when perfectly dry, it will keep vigorous for a long time.

"The frugal housewife, says *Edlin,* who would enjoy the luxury of eating good unadulterated bread, "ought to be possessed of such directions, as that, with little labour or trouble, she may soon learn to grind her own wheat, separate the flour from bran, make it up and bake it into bread. To captains of ships, to military men, and to such as travel into unfrequented regions, where if any bread is to be procured, it is in general execrable. The baker, whose habits and education do not lead him to investigate speculative doctrines and opinions, may find several observations that will prove serviceable in the prosecution of his business," by an attention to such facts and hints as have been recorded.

"Impressed with this sanguine hope, we anticipate the period when we shall see he art of bread making, instead of forming a confused and unintelligible article in our Encyclopœdias and Dictionaries, attain a proper rank among the liberal sciences. But to these ends, and also a knowledge of the nature and ready procuration as well as best application of yeast is requisite.

Some whisk yeast well until it becomes thin, then lay it upon a dry platter or dish repeatedly, with a soft brush, as hinted by Mr. Cooke; the top is turned downwards to keep out the dust, but not the air, which is to dry it. By this method it may be continued till it be two or three inches thick, when it may be preserved in dry tin cannisters for a long time good. When used for baking, a piece is to be cut off, and laid in warm water to diffuse or dissolve, when it is fit for use.

The principle upon which yeast operates in the panary fermentation, is to be referred in a great measure to the quantity of carbonic acid or fixed air, which it generates and contains. So that in baking very light cakes, which are are held as very delicate by some, snow water has been used, or rather snow itself; there is no doubt but that the pores or flocculi of snow contain a considerable quantity of fixed air.

Take two ounces of flour, boil it over the fire in a quart of water, till it comes to the consistence of a thin jelly, and pour it into the middle part of Dr. Nooth's machine for impregnating water with fixed air; then put into the lower vessel some coarse powdered marble, and pour on it sulphuric acid diluted with water. The apparatus is now to be adjusted, and the upper vessel put into its place, and nearly stopped. The carbonic acid, now extricated, passes thro' the valve, and ascends into the middle and upper part of the machine, where the gas is absorbed by the flour jelly, in considerable quantity, and in the course of a few hours the matter will be found so strongly impregnated, as to be in a state of fermentation. This artificial yeast may now be removed from the machine, and put into a bottle for use. This may be made in situations where it is impossible to procure yeast from a Brewhouse. Mixed with dough, the ingenious Mr. Henry (of Manchester) asserts, it answers every purpose of brewer's yeast.

Thicken two quarts of water with four ounces of fine flour, boil it for half an hour, then sweeten it with three ounces of brown sugar; when almost cold, pour it, along with four spoonfuls of baker's yeast, into an earthen jug, deep enough for the fermentation to go on without running over; place it for a day near the fire, then pour off the thin liquor from the top, shake the remainder, and close it up for use, first straining it through a sieve. To preserve it sweet, set it in a cool cellar, or hang it some depth in a well. Keep always some of this to make the next quantity that is wanted.

From what has been said in Mr. Henry's process for a substitute for yeast, it must be obvious, that any of the mineral waters impregnated highly with carbonic acid might be a valuable auxiliary. Whether fermented liquors have been ever used with this view, as cyder or bottled small beer, we know not. Mr. Edlin recommends half a pound of fine flour, half a pound of brown sugar, and a quarter of a peck of bruised malt, to be boiled over the fire a quarter of an hour, in half a gallon of water; then strain the liquor through a sieve into an upright jug, and when cooled to 80°, a pint of artificial or fictitious Seltzer water may be added. (Soda water highly impregnated with carbonic acid will answer every purpose.) It will soon begin to ferment; it should then be set before the fire, and when the ebullition ceases, the yeast will sink to the bottom, and the clear liquor is to be poured off, when it will be fit for use. In this way yeast may be made from water, impregnated with fixed air from the waters of Ganesse, near Paris; from the Pyrmont, Spa, and Seltzer mineral aerated springs. The waters of Saratoga, in the province of New York, in America, and from various other natural mineral acidulæ or aerated mineral springs.

The honourable Captain Cochrane mentions the following, as the method of making yeast, practiced by the bakers of Edinburgh.

Take two ounces of hops, boil them for an hour in two gallons of water, and while boiling hot, scald 10lbs. of flour, and stir it very well into a paste; do this about eleven o'clock in the forenoon, let it stand till six o'clock in the evening, then add about a quart of yeast to forward the fermentation, and mix them well together. Next morning, add as much more flour and water as will be sufficient to make it into a dough, and in the afternoon it will be fit for setting sponge and baking. Reserve always a piece of old dough to mix with the new batch instead of the yeast, which is necessary only the first time, to hasten the process. The above quantity will suffice for 120 quartern loaves. This process in Scotland requires about thirty hours, but in a warm climate a few hours would suffice, as fermentation there advances with great rapidity; a due attention must be paid to that circumstance, as every thing depends upon it.

In this method of making yeast will be wanted boiler, cooler, vats, and indeed all the apparatus necessary for a brewery. Then take four bushels of the best malt, ground as for beer, mash it in the same manner as the brewers do, with 62 gallons of water, at the temperature of 180°; let it be close covered up for two hours, then draw the liquor clear off, and pour on the same quantity of water upon the grains a second time, at nearly the boiling point; let this stand for an hour, then draw it off, and mix it in your coolers with the first wort, and when it is about blood warm, add four quarts of yeast to produce the fermentation, and after it has began to ferment the first time, (the froth running out into a receiver for the purpose) throw it back again, and when it has fermented again, throw it back a second time, and it will then, after the third fermentation, be fit for use, as will be perceived by its being of the the thickness that good yeast ought to be. Four bushels of malt made in this way, produce about twenty-four quarts of yeast. It is an expensive and troublesome way of procuring it, but Mr. Gillespie finds that a quart of it will go as far as a gallon of distiller's yeast. He at first, in order to lessen the expense, endeavoured to make the refuse liquor into beer, but found it was so much impoverished as not to answer this purpose; it is not however entirely wasted, as he disposed of it to the vinegar makers, who gave him a trifle for it, which may perhaps pay the expense of fuel and labour.

Boil potatoes of the meally sort until they are thoroughly soft, skin and mash them very smooth, and put as much hot water on them as will make a mash of the consistence of common small beer yeast, but not thicker. Add to every pound of potatoes two ounces of treacle, and when just warm, stir in for every pound of potatoes two large spoonfuls of yeast. Keep it warm till it has done fermenting, and in twenty-four hours it will be fit for use. A pound of potatoes will make near a quart of yeast, and when made it will keep three months. This yeast has been found to answer so well as not to leave it possible for any one to distinguish the bread made with it from that made with brewer's yeast.

It is not known, says Tissot, in his Essay on bread corn and bread, to what country we are originally indebted for wheat; but when we reflect, that it furnishes man in most civilized countries with a very considerable portion of his food, it is a happy circumstance that it can accommodate itself to so great a variety of climates. Hence, it is found in all Europe, in Egypt, and throughout Barbary, that is to say, in all the most populous parts of Africa; in Mexico, in the most flourishing provinces of Peru, and in those of North America. Wheat is also the food of man in many parts of Persia and Hindostan; and even in China, a rice country, wheat is in daily use in most of its provinces.

